# Evaluation of the Impact of a Novel Visual Training Video Game on Oculomotor Function and Visual Symptoms in Subjects with Parkinson’s Disease and Convergence Insufficiency: A Pilot Study

**DOI:** 10.3390/life16050825

**Published:** 2026-05-15

**Authors:** David P. Piñero, Carla Pérez-Casas, Alba Pina-Balofer, Carmen Bilbao, Carlo Cavaliere-Ballesta, Laurent Bataille, Rafael J. Pérez-Cambrodí

**Affiliations:** 1 Department of Optics, Pharmacology and Anatomy, University of Alicante, San Vicente del Raspeig, 03690 Alicante, Spainrjcambrodi@gmail.com (R.J.P.-C.); 2 Department of Ophthalmology, Vithas Medimar International Hospital, 03016 Alicante, Spain; 3 Visitrain S.L., Science Park, University of Alicante, 03005 Alicante, Spain; apina@visitrain.onmicrosoft.com (A.P.-B.); ccavaliere@visitrain.onmicrosoft.com (C.C.-B.); lbataille@visitraintherapies.com (L.B.); 4 Department of Applied Physics, Faculty of Science, University of Zaragoza, 50009 Zaragoza, Spain

**Keywords:** Parkinson’s disease, convergence insufficiency, visual rehabilitation, digital therapy, NSUCO, King-Devick, CISS

## Abstract

Rationale and objectives: Parkinson’s disease (PD) significantly affects visual function, especially convergence and eye movements, impacting tasks such as reading. The objective was to investigate preliminarily the impact of the use of digital visual training in PD patients with associated convergence insufficiency (CI). Materials and methods: Pre–post pseudo-experimental pilot study to evaluate the impact of a novel digital therapy system (video game for use on a mobile phone or tablet) in 13 patients with PD and CI, with a mean age of 67 years. A comprehensive visual assessment was performed before and after a 6-week home-based visual rehabilitation, including measurement of near point of convergence (NPC), near positive fusional vergence (PFV), oculomotor tests (NSUCO and King-Devick tests), and symptom assessments with two validated questionnaires (CISS and SQVD). Results: Treatment adherence was variable, ranging from 0.8% to 124.7%. Despite this, significant improvements were found after therapy in break (*p* = 0.022) and recovery points of the NPC (*p* = 0.007), as well as break (*p* = 0.003) and recovery points in near PFV (*p* < 0.001). In the NSUCO test, the total score improved significantly from 23.9 ± 4.2 to 26.2 ± 3.7 after therapy (*p* = 0.003). Furthermore, a significant reduction in the total King-Devick test time was observed, decreasing from 79.4 ± 28.8 s to 69.0 ± 21.5 s with therapy (*p* = 0.034). Finally, symptom questionnaire scores also decreased significantly with therapy (CISS *p* = 0.037, SQVD *p* < 0.001). Conclusions: The digital vision therapy system evaluated seems to improve oculomotor control and reduce visual symptoms associated with CI in PD patients. Studies with larger sample sizes and a control group are needed to fully validate the therapeutic effectiveness of this tool.

## 1. Introduction

Parkinson’s disease (PD) is a neurodegenerative disorder of the nervous system that evolves in a chronic and progressive manner, meaning that its symptoms persist and worsen over time. Currently, it is the second most common neurodegenerative disease after Alzheimer’s disease and belongs to the group known as “Movement Disorders” [1,2]. It is characterized by the degeneration of neurons in the substantia nigra of the brain (midbrain), which leads to a decrease in dopamine production, a key neurotransmitter for motor coordination. This lack of dopamine results in typical motor symptoms such as resting tremors, muscular rigidity, and bradykinesia (slowness of movement) [1,2].

Multiple visual disturbances have been observed, including decreased visual acuity [3], contrast sensitivity [4], color perception [5], and motion perception [6], as well as abnormalities in pupillary reactivity [7] and eye movements [8]. These deficits affect both the speed of visual processing and visuospatial orientation, as well as facial recognition. Among the most frequent symptoms are difficulty reading, double vision, illusions, and sensations of “presence” or “passage,” which can be considered as hallucinations [6]. In the preclinical stage, autonomic system dysfunctions may occur, altering pupillary reaction, color vision, and stereopsis, alongside postural instability and defects in eye tracking and visuomotor adaptation [6]. Deterioration progresses in dementia associated with PD, worsening oculomotor, visuospatial disturbances, and visual hallucinations [6].

Among the visual problems associated with PD is convergence insufficiency (CI), which is a binocular vision disorder characterized by difficulty maintaining fusion while looking at a near target due to a tendency of the eyes to drift outwards [9]. Typical symptoms linked to CI include eye strain (asthenopia), double vision (diplopia), headaches, blurry vision, shifting or wobbly text while reading, and difficulty understanding what has been read [10]. Additional noticeable symptoms that appear after brief reading sessions or extended close-up work include drowsiness and a lack of focus [10]. Logically, all of these symptoms have a very significant impact on the patient’s quality of life [11]. In PD, the prevalence of CI is especially significant. Irving and colleagues [12] assessed CI in 80 individuals with PD alongside 80 healthy controls. They reported a CI prevalence of 43.8% in the PD group, compared to 16.3% in the control group. Notable symptoms (scoring ≥21 on the CISS-15) were observed in 53.8% of PD patients versus 18.8% of controls. In a more recent study, Herrero-Gracia and collaborators [13] compared 71 PD patients with 97 controls and found that the rate of CI increased markedly with more advanced Hoehn and Yahr stages (41% in PD participants vs. 12% in controls; stage-specific odds ratios: 1.67 for stage I, 1.27 for stage II, and 2.36 for stage III; *p* < 0.007).

Vergence/accommodative therapy is the option with the greatest scientific support for managing convergence insufficiency [14], although studies in subjects with neurodegenerative diseases are very scarce. Specifically, only two studies have reported some results of vision therapy for treating CI in PD patients, based on combining office-based sessions with home reinforcement [15,16]. The main limitation identified in these two studies is the considerable difficulty in achieving treatment adherence and compliance, with high dropout rates as soon as therapy begins [15,16]. Perhaps this could be addressed by using video games on mobile phones or tablets, allowing patients to undergo therapy at home while the therapist monitors their progress online [17]. It must be considered that many patients with PD have mobility limitations, presenting difficulties for continuous and periodic travel to in-person visual training appointments. The aim of the current study was to preliminarily investigate the impact of the use of a novel digital visual training system in PD patients with CI, evaluating changes in clinical signs and symptoms associated with this binocular vision condition.

## 2. Materials and Methods

### 2.1. Patient Selection

A prospective, non-comparative pre–post pseudoexperimental study was conducted at the facilities of the Alicante Parkinson’s Association (Alicante, Spain). The study adhered to the principles of the Declaration of Helsinki and was approved by the Ethics Committee of the Department General University Hospital of Alicante (code CEIm: 2025-022) and the Spanish Agency of Medicines and Medical Devices (1531/25/EC-R).

Participants included in the study were required to have a confirmed diagnosis of PD, based on the clinical criteria of the Movement Disorder Society (MDS) [18]. These criteria establish the mandatory presence of bradykinesia as a cardinal symptom, accompanied by at least one of the following signs: rest tremor with a frequency between 4 and 6 Hz, muscle rigidity manifested as resistance to passive movement, or postural instability that cannot be attributed to visual, vestibular, or cerebellar causes [18]. Furthermore, only patients in mild to moderate stages of the disease, according to the Hoehn and Yahr scale, were included [19]. Specifically, those deemed eligible were individuals with unilateral symptoms (stage 1), bilateral symptoms without impairment of balance (stage 2), or bilateral symptoms with mild postural instability, provided that functional independence is preserved (stage 3). Another inclusion criterion was pharmacological stability. Participants were required to have maintained a stable regimen of antiparkinsonian treatment, such as levodopa or dopaminergic agonists, for at least three months prior to the start of the study, without significant medication adjustments during this period. Finally, an adequate cognitive level was considered essential for participation in the required tasks. For this purpose, the Montreal Cognitive Assessment (MoCA) was administered in its Spanish-validated version. A cut-off score of ≥18 points was established, a value considered compatible with the ability to understand instructions, follow the protocol autonomously or with minimal assistance, and participate reliably in the visual training process [20].

From a visual standpoint, inclusion criteria included participants with visual acuity equal to or better than 0.2 LogMAR (equivalent to 20/32), ensuring sufficient functional vision to accurately perform the study’s visual tests. Likewise, a subjective refraction within the range of ±1.00 diopter (D) of the participant’s habitual correction was required, allowing for the selection of individuals with adequate refractive compensation for both distance and near vision. This broad threshold is justified by the visual particularities of advanced age, where accommodation is considerably reduced, without this interfering with the vergence functions or stereoacuity being evaluated. In addition, the diagnosis of CI was confirmed, considering as reference the average cutoff values described in the systematic review by Cacho et al. [21], considered a benchmark in the diagnostic criteria for this condition: difference in exophoria between distance and near ≥4∆, being exophoria greater at near, NPC break point of 6 cm or greater, PFV break point at near of ≤15∆, and a score of 16 or higher on the Convergence Insufficiency Symptom Survey (CISS) questionnaire.

Exclusion criteria included patients unable to adequately understand or perform the clinical tests during the initial assessment, cases of CI associated with brain injury or other neurological diseases, and patients who did not initiate the vision therapy treatment.

### 2.2. Examination Protocol

The study was organized into two visits. At the baseline visit, each patient was evaluated and the inclusion and exclusion criteria were verified. The investigator confirmed the patient’s suitability by collecting the following data from the anamnesis: confirmed diagnosis of PD according to the clinical criteria established by the MDS, PD stage classified as 1 to 3 according to the Hoehn and Yahr scale, at least 3 months on a stable regimen of antiparkinsonian medication, type of refractive correction used, history of ocular surgeries, presence of ocular pathologies (glaucoma, retinal detachment, diabetic macular edema, etc.), and relevant systemic conditions such as diabetes, hypertension, etc. The selection of Hoehn and Yahr stages 1–3 ensures that participants have sufficient ability to collaborate in the visual assessments and comply with the therapy protocol. Pharmacological stability minimizes variability in motor symptoms. Once these aspects have been verified, a complete visual examination was carried out to determine whether the diagnostic criteria for symptomatic CI were met and whether a visual training program could be prescribed. Specifically, the following tests were performed:

- Measurement of distance visual acuity without and with correction (5 m) using test ETDRS chart.
- Manifest refraction.
- Measurement of near visual acuity without and with correction (40 cm).
- Worth test at distance (6 m) and near (40 cm): this was used to determine if there was unilateral suppression under binocular conditions.
- Measurement of ocular deviation at near and distance using the cover test.
- Measurement of NPC: Fusion break and recovery points were measured using the Lang bar. The patient was asked to report when the Lang bar’s target first appeared double as the bar was moved toward their eyes (break point), and then when it became single again as the bar was moved away (recovery point). This procedure was performed three times, and the average of the three measurements was recorded.
- Measurement of fusional vergences at near (40 cm): Base-in (divergence) and base-out (convergence) fusional vergences were assessed using a prism bar. Two key points were recorded: the break point (when double vision first appeared), and the recovery point (when single vision was restored). This testing was performed under photopic lighting conditions. The patient was asked to maintain the fixation to an optotype corresponding to a visual acuity of 0.2 logMAR.
- Measurement of stereopsis using the TNO test (Laméris, Ede, The Netherlands).
- Evaluation of the symptomatology with two validated questionnaires: Convergence Insufficiency Symptom Survey (CISS) [22] and Symptom Questionnaire for Visual Dysfunctions (SQVD) [23].
- Assessment of oculomotricity with the NSUCO (Northeastern State University College of Optometry’s Oculomotor) test [24]: This is a standardized test with established scoring criteria used to characterize pursuit and saccadic eye movements [19]. During the test, a trained examiner subjectively assesses eye movements—both smooth pursuit and saccades—across four performance areas: ability, accuracy, head movement, and body movement [24]. The patient sat facing the examiner, who performed the test binocularly. Small colored spheres (0.5 cm in diameter) mounted on a rod were used as fixation targets and presented at a distance of 40 cm. To evaluate smooth pursuit movements, the stimulus was moved in a circular pattern (approximately 20 cm in diameter) both clockwise and counterclockwise. To assess saccadic movements, the patient was asked to alternate fixation between two stimuli placed 20 cm apart horizontally [24]. The scoring criteria used for this test were as follows:
  ○ Smooth pursuits:
    ▪ Patient’s ability to perform two rotations (ability):
      ➢ 1 point: Half rotation not completed;
      ➢ 2 points: Half rotation;
      ➢ 3 points: 1 rotation in each direction;
      ➢ 4 points: 2 rotations in one direction;
      ➢ 5 points: 2 complete rotations.
    ▪ Patient’s ability to perform two rotations without refixations (accuracy):
      ➢ 1 point: More than 10 refixations;
      ➢ 2 points: 5 to 10 refixations;
      ➢ 3 points: 3 to 4 refixations;
      ➢ 4 points: 2 refixations or less;
      ➢ 5 points: No refixations.
    ▪ Patient’s ability to perform two rotations without head or body movements:
      ➢ 1 point: Exaggerated body or head movement;
      ➢ 2 points: Large or moderate movement;
      ➢ 3 points: Slight movements but constant;
      ➢ 4 points: Slight movements but intermittent;
      ➢ 5 points: No head or body movement.
  ○ Saccadic movements
    ▪ Patient’s ability to perform 5 cycles of change in fixation between the two stimuli presented (ability):
      ➢ 1 point: 1 cycle or no ability;
      ➢ 2 points: 2 cycles;
      ➢ 3 points: 3 cycles;
      ➢ 4 points: 4 cycles;
      ➢ 5 points: 5 cycles.
    ▪ Patient’s ability to perform 5 cycles of change in fixation without correcting refixations (accuracy):
      ➢ 1 point: Very significant hyper- or hypometric movements;
      ➢ 2 points: Large to moderate hyper- or hypometric movements;
      ➢ 3 points: Slight hyper- or hypometric movements but constant;
      ➢ 4 points: Slight hyper- or hypometric movements but intermittent;
      ➢ 5 points: No correcting refixations.

Evaluation of saccadic performance with the King-Devick (K-D) test: used to evaluate the quality of saccadic eye movements, as well as attention and visual processing. It is based on the rapid and accurate reading of series of numbers arranged on cards. Specifically, it consists of three phases: a practice card to familiarize the patient with the task, followed by three main cards with a progressively more complex arrangement of numbers that must be read from left to right as quickly as possible. During the main phase, the time taken for each card is recorded and any errors made are noted. The sum of the times provides the total reading time, which, together with accuracy, allows for the assessment of oculomotor performance. A prolonged time or a greater number of errors may indicate difficulties in saccadic control or visual processing [25].

Once the basal examination was completed and the diagnosis of CI confirmed, the patient was informed of the need for visual therapy and what the procedure entails. Since many patients do not have adequate mobility and may have some difficulty attending in-person visual training visits, training was prescribed at home using the Visitrain VG system (Visitrain S.L., Alicante, Spain), which could be installed on the patient’s mobile phone or tablet without the need for complex technology. It was always used with a Wi-Fi internet connection. The app was then installed on the subject’s electronic device, and the patient was trained to understand the therapeutic game and how to interact with it. They were prescribed a 10 min daily session with one resting day per week for 6 weeks. Training data was transferred to the cloud after each session, allowing the examiner to remotely monitor therapy compliance levels and improvements in the scores obtained. A contact mobile phone number was provided for them to call with any questions or technical issues during the six weeks of training.

A complete visual examination was performed after finishing the 6-week training period, including the evaluation of the following parameters:

- Worth test at distance and near.
- Measurement of ocular deviation at near and distance using the cover test.
- Measurement of break and recovery NPC points.
- Measurement of near fusional vergences at near (including break and recovery) using a prism bar.
- Measurement of stereopsis using the TNO test.
- Evaluation of the symptomatology with the CISS and SQVD questionnaires.
- Evaluation of oculomotricity with NSUCO and K-D tests.

The scoring obtained during the game, total duration of therapy completed, and compliance level were also registered and analyzed.

### 2.3. Visual Therapy App: Visitrain VG

**Figure 1:**
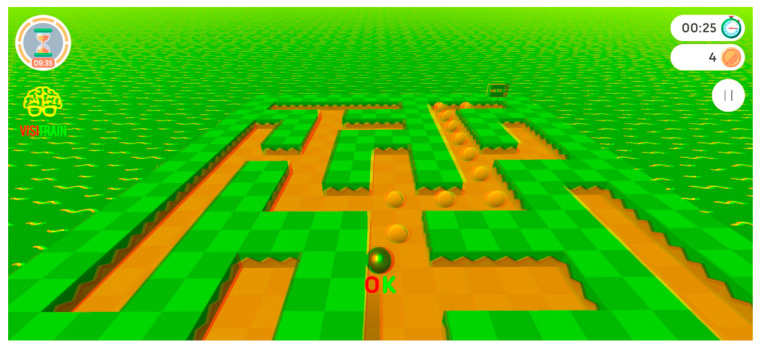
Screenshot of the Visitrain VG mobile video game (Visitrain S.L.).

Visitrain VG is a clinically validated tool for treating CI through red–green dissociation [17] (Figure 1). It offers an interactive, game-like environment in which patients can improve their convergence skills in an enjoyable manner, while also enhancing the coordination of binocular saccadic eye movements. The program includes various challenges and rewards to keep patients engaged. One of its key advantages is that it runs on any Android or iOS device—smartphone or tablet—eliminating the need for specialized equipment. The system incorporates several features to support effective rehabilitation:

➢ Dissociated visual environments that work with different filter types to create convergence demands, enabling patients to practice this specific skill.
➢ An immersive gamified setting where the device interacts with the patient in a novel way, helping them feel part of the environment and motivating them to continue progressing through the game.
➢ Suppression control mechanisms that detect and manage when a patient’s brain might be suppressing input from one eye.
➢ Continuously moving adventure maze scenarios that provide ongoing stimulation of binocular saccadic function.
➢ Attention-grabbing stimuli, including simultaneous presentation of visual targets and discrimination tasks within complex backgrounds.
➢ Participants’ interaction with the task using the gyroscope of the electronic device.
➢ Use of an adaptive psychophysical method for adjusting the level of dissociation introduced in the game according to the patient’s responses and failures. Specifically, an adaptation of the PEST (Parameter Estimation by Sequential Testing) [26] method is used, the aim of which is to work close to the threshold following a specific set of rules, promoting its progressive improvement.

Patient adherence is tracked in real time via a cloud-based platform, which also allows the clinician to analyze and review therapy progress remotely.

### 2.4. Statistical Analysis

Data collection and analysis were performed by an independent researcher. Statistical analysis was carried out using SPSS version 24.0 (IBM, Armonk, NY, USA). For every quantitative variable, descriptive measures such as the mean, median, standard deviation, and range were calculated. The Shapiro–Wilk test was used to determine whether the data followed a normal distribution. To compare differences between pre- and post-visits, paired Student’s *t*-test (for normally distributed data) and the Wilcoxon test (for non-normally distributed data) were applied. The result was considered statistically significant when the *p*-value fell below 0.05. *p*-values were adjusted with the Benjamini–Hochberg procedure to control the risk of type I error due to the multiples comparisons done.

## 3. Results

A total of 42 patients from the Alicante Parkinson’s Association were screened, of whom only 25 (59.5%) met the diagnostic criteria for CI. Of those, 12 did not wish to participate in the study. A sample of 13 patients with a mean age of 67.1 years (standard deviation: 10.1; median: 69.0; range: 40 to 80 years old) was finally recruited. Mean basal monocular corrected distance visual acuities (CDVA) were 0.10 ± logMAR in both eyes. Table 1 summarizes all these outcomes related to the game performance and compliance with treatment. No correlation was observed between age and the level of treatment compliance (r = −0.041, *p* = 0.894).

**Figure 2:**
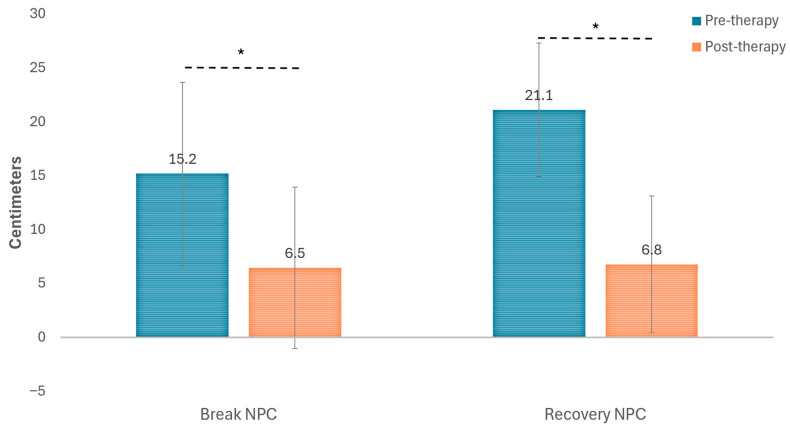
Changes in break and recovery of the near point of convergence (NPC) with the digital visual training program. The asterisk is indicating those changes that reached statistical significance.

**Figure 3:**
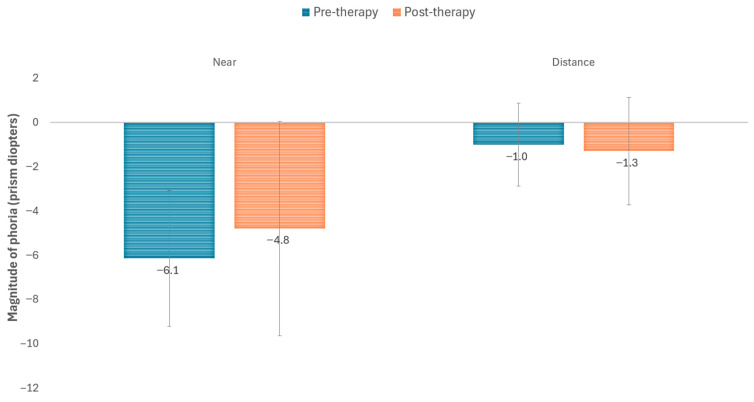
Changes in the magnitude of the near and distance phoria with the digital visual training program.

Figure 2 and Table 2 display changes occurring in NPC with the visual training program. As shown, a statistically significant reduction was observed in break (*p* = 0.022) and recovery NPC points (*p* = 0.007). However, no significant modifications were found in the magnitude of the deviation at distance (*p* = 0.336) and near (*p* = 0.279) measured with the cover test (Figure 3). Moderate correlations were found between the level of compliance and the reduction achieved with therapy in break (r = −0.478, *p* = 0.137) and recovery (r = −0.524, *p* = 0.183) NPC points, although these correlations did not reach statistical significance.

Table 2 summarizes the results obtained in terms of NPC and fusional vergences measured at near before and after therapy. Significant improvements were found following therapy in the break (*p* = 0.003) and recovery points (*p* < 0.001) of PFV, whereas no significant changes were found in the break (*p* = 0.108) and recovery points (*p* = 0.126) of negative fusional vergence (NFV). Poor and non-statistically significant correlations were found between the level of compliance and the increase achieved with therapy in break (r = 0.067, *p* = 0.864) and recovery (r = 0.336, *p* = 0.376) PFV points measured at near.

Table 3 shows the outcomes obtained after and before therapy in the NSUCO and K-D tests. In the NSUCO tests, a significant improvement in the score for pursuit (*p* = 0.006) and saccadic movements (*p* = 0.008) was obtained as well as in the total NSUCO score (*p* = 0.003). A trend of a reduction in the time needed to read the numbers from the three cards of the test was observed. However, those changes only reached statistical significance for the time required to read the first card (*p* = 0.018) as well as for the total time required to do the K-D test (*p* = 0.034).

**Figure 4:**
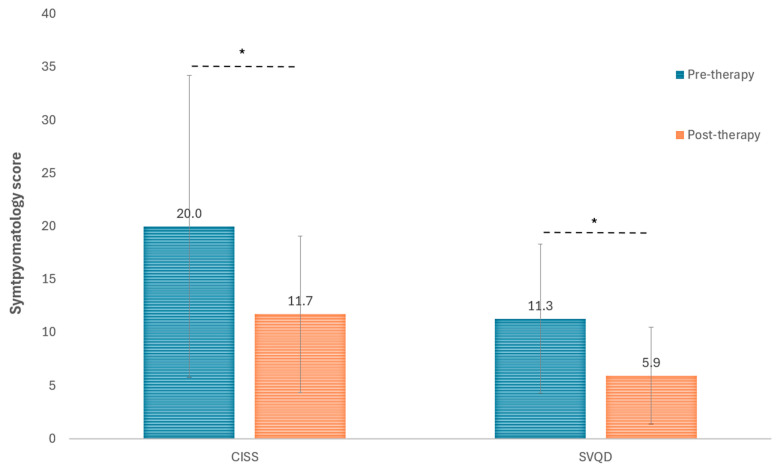
Changes with the vision therapy program in the symptomatology scores obtained with the CISS and SQVD questionnaires. The asterisk is indicating those changes that reached statistical significance.

Significant improvements were also found in symptomatology, with a significant decrease in the two validated questionnaires used for this purpose, CISS (*p* = 0.037) and SQVD (*p* < 0.001) (Figure 4). Concerning stereopsis, it did not experience significant changes with the visual training program performed (*p* = 0.402). Specifically, mean stereoacuity changed from 612.0 (standard deviation: 731.5; median: 240.0; range: 120 to 1980 s of arc) before therapy to 540.0 (standard deviation: 657.7; median: 240.0; range: 60 to 1980 s of arc) after therapy.

**Figure 5:**
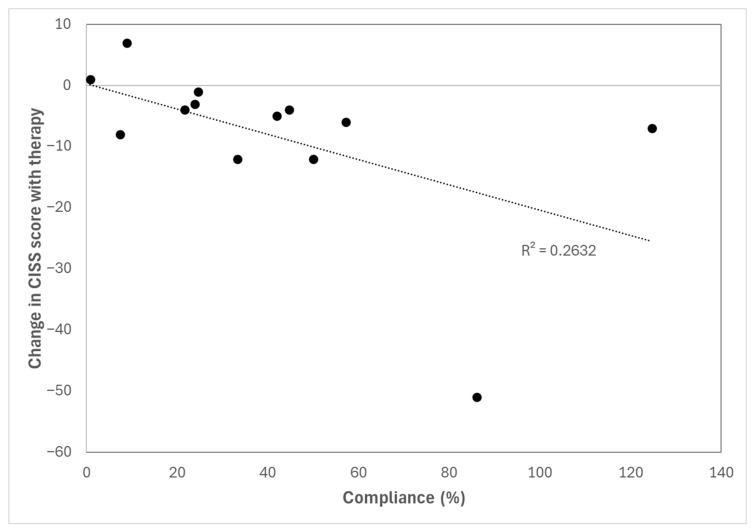
Relationship between the symptomatology score obtained with the CISS questionnaire and the level of compliance with the therapy.

Regarding the relationship between the level of adherence to therapy and the change in the evaluated parameters, moderate to good and statistically significant correlations were found between adherence and the change in the CISS score (r = −0.606, *p* = 0.028) (Figure 5), the total time of the K-D test (r = −0.733, *p* = 0.004), and the break NPC point (r = −0.547, *p* = 0.043). In contrast, poor or very poor correlations were found between adherence and the change in near break PFV point (r = 0.323, *p* = 0.362), NSUCO score (r = 0.277, *p* = 0.360), and SQVD score (r = −0.097, *p* = 0.753).

## 4. Discussion

This study demonstrates the potential usefulness of a digital vision therapy system for the management of CI in patients with PD. The use of 10 min home-based playing sessions over one and a half months induced significant improvements in various signs associated with CI, as well as in symptoms. This suggests that even a relatively limited dose of training can have a positive impact on patients with PD, possibly due to the residual neuroplasticity that can be harnessed through repetitive visual stimulation. Magrinelli et al. [27] highlight that, although PD involves marked dopaminergic loss, some synaptic plasticity is still preserved. This supports the use of repetitive and multisensory therapies, as they can activate neuronal adaptation mechanisms and enhance functional recovery through the reorganization of motor circuits [27].

### 4.1. Changes in NPC and near Fusional Vergences

Clinically, in our series, significant reductions were observed following the visual training program in the break and recovery points of NPC, indicating a greater ability to maintain ocular alignment at very close distances. This was associated with a significant increase in the break and recovery points of PFV at near, confirming that the ocular alignment at close distances was maintained with stability and with sufficient reserves to compensate for any potential instability. These results are consistent with a study conducted by Álvarez et al. [28], who also found a significant reduction in NPC in neurologically healthy adults with CI following the application of office-based vision therapy with home reinforcement. Furthermore, these authors also showed a notable increase in the amplitude of PFV, both in break and recovery values [28]. In addition to the clinical improvements, the study by Álvarez et al. [28] incorporates an additional component by analyzing functional brain changes using functional magnetic resonance imaging. These authors found increased activity in brain regions involved in vergence control, such as the dorsolateral prefrontal cortex, the cerebellum, and the brainstem following vision therapy. These changes suggest that rehabilitation not only acts at the ocular level but also induces adaptations in neural processing that support the improvement of binocular function. Kergoat et al. [15] documented two individual cases of patients with PD and CI who completed conventional orthoptic therapy. Both cases showed improvements in NPC, PFV, and reported symptoms, suggesting that the intervention may be beneficial. In any case, comparison with the previous literature must be done with care, as most studies on CI training involve a higher number of sessions and more time dedicated to the exercises. In patients with PD [14,15,28], these more intensive protocols are not feasible, considering the difficulty of such patients attending in-person visits and the tiredness when the training sessions are prolonged.

Besides the improvement in PFV, a significant improvement following vision therapy was also observed in NFV, although divergence reserves are not typically affected primarily in CI. These findings were consistent with the results of the study previously conducted by Scheiman et al. [29], in which a significant improvement in NFV following vergence/accommodative therapy was also demonstrated, both clinically and through objective eye movement recordings. It should be noted that the visual therapy protocol used in that study also included training of divergence reserves, in contrast to the system used in the present study, which is primarily focused on convergence training. These authors concluded that, despite clinical NFV measurements appearing greater than normal, subjects with symptomatic CI may have deficient NFV when measured with objective eye movement recordings [29].

### 4.2. Changes in the Magnitude of the Phoria

Regarding the magnitude of the phoria measured with the cover test, no statistically significant differences were observed in our series, indicating that the angle of deviation did not change substantially following therapy. This was expected, since vision therapy is not aimed at modifying the magnitude of the deviation, but rather at improving vergence flexibility and fusion capacity. These results contrast with the findings of the study by Schulman et al. [30], in which a statistically significant reduction in near exodeviation (2.6Δ on average; *p* < 0.001) was demonstrated following therapy in children aged 9 to 14 years with CI. Given that Schulman et al. focused on a pediatric population with a still-developing visual system, their results are not directly comparable to those obtained in our adult sample with PD, whose neuroadaptive response may differ. In contrast, previous studies on the use of vision therapy in patients with PD and CI did not demonstrate either clinical or statistically significant changes in near phoria following vision therapy [15,16]. Besides this, other studies have also reported successful outcomes with different varieties of vision therapy in adults with CI, but without neurological disorders [31,32,33].

### 4.3. Changes in Oculomotricity

As no objective measures of ocular motility, such as eye tracking, were recorded in the present pilot study, two different subjective tests were employed: the NSUCO and King-Devick (K-D) tests. NSUCO scores showed significant improvements in both pursuits and saccades, with an overall increase in the total score, reflecting an improvement in the quality, accuracy, and control of voluntary eye movements, which are essential for reading tasks and visuomotor coordination. Likewise, in the KD test, significant improvements were observed in the total score and in subtest I, while subtests II and III, as well as the number of errors, did not show significant changes. The reduction in total reading time indicated greater visual efficiency following therapy, albeit with some inter-subject variability, possibly linked to the degree of motor and cognitive impairment in PD. These results are supported with those reported by Lin et al. [34], who found that the K-D test is sensitive to changes in the speed and accuracy of saccadic movements in patients with PD. Specifically, these authors found a mean total K-D score of 66 s in the PD group, which was significantly higher than the mean value in age-matched healthy subjects (52 s) [34]. In the present study, the mean total K-D time in the PD sample was 72 s, which is comparable to the value reported in the aforementioned study.

To our knowledge, this is the first study reporting the outcomes of NSUCO test in patients with PD. The NSUCO is a simple and rapid clinical tool whose scoring has demonstrated good inter-rater and test–retest reliability in experienced examiners [24,35]. Recent work has shown significant correlations between NSUCO scores and objective eye-tracking parameters, supporting its validity as a surrogate for instrument-based assessment of saccadic eye movements [36]. Moreover, the NSUCO can be administered in just a few minutes, which is particularly advantageous in this population, where lengthy and demanding assessment batteries may increase fatigue and reduce cooperation. In the present study, the same experienced examiner performed all the NSUCO evaluations, avoiding potential inter-examiner bias.

In the present study, the improvement in vergence function following vision therapy for CI was then associated with an improvement in oculomotor performance. It should be noted that the digital training system used provides stimuli for the simultaneous training of pursuit and saccadic movements, alongside the enhancement of vergence amplitude. Given that fluent reading requires precise coupling between saccadic and vergence eye movements, the improvements in convergence and vergence ranges observed in our patients may reasonably be expected to translate into better binocular coordination during reading and a reduction in re-reading behavior, as previously described in patients with CI [37]. Gaertner et al. [38] evaluated how vergence deficits affect binocular coordination of saccadic movements and fixation during reading in children. The authors found that, although saccade amplitude did not differ significantly between children with vergence deficits and controls with normal orthoptic function, the former presented greater binocular misalignment during and after saccades, indicating poor binocular coordination. After orthoptic training, children significantly improved this coordination, suggesting an adaptive interaction between the saccadic and vergence systems [38]. A study conducted by Hirota et al. [39] on patients with intermittent exotropia of CI type showed that a greater number of involuntary re-readings was directly related to greater binocular saccadic misalignment during reading. This correlation indicates that binocular misalignment (CI type) in saccadic movements hinders reading fluency. Scheiman et al. [40] reported improvements in reading comprehension and reading composite scores following office-based vergence/accommodative therapy, with the greatest improvements in those who responded early to treatment. However, one year later, a randomized controlled trial [41] demonstrated that office-based vergence/accommodative therapy was no more effective than office-based placebo therapy in improving reading performance in children aged 9 to 14 years with symptomatic CI following 16 weeks of treatment. It should be noted that students in the placebo group of that study may have received supplemental reading intervention or tutoring at school, thereby highlighting the difficulty in controlling all confounding factors when evaluating the impact of vision therapy on reading performance. In any case, the available scientific evidence supports the relationship between vergence and oculomotor function within a vision therapy program, which would also account for the significant improvement in symptomatology observed in our series, particularly in symptoms related to reading performance and difficulties.

It should be emphasized that reading and other near-vision tasks are among the most important daily activities for many people with PD and that reading difficulties are highly prevalent and have a substantial impact on their everyday life [42,43]. For this reason, the improvement in oculomotricity and vergence systems with the digital training applied seems to be a crucial factor contributing to the subjective report of patients improving in reading.

### 4.4. Changes in Symptomatology

Regarding the questionnaires used to assess CI symptoms, on the one hand, the CISS showed a significant reduction in symptoms, reflecting a clear subjective perception of visual improvement by the patients. Likewise, the SQVD also showed a significant improvement, evidencing that the therapeutic improvements were also reflected in daily life as reported by the patients themselves. In line with these findings, a recent study by Abdi and Kangari [44] evaluated the validity and reliability of the CISS questionnaire specifically in an adult population with presbyopia. It was confirmed that the questionnaire is a valid tool for discriminating between subjects with CI and those with normal binocular vision. However, the results of that study suggested that the questionnaire could assess different symptomatic aspects beyond CI itself. These findings support the utility of the CISS as a subjective tool in presbyopic patients, but also underscore the need to complement it with objective clinical tests [45]. In the current study, two validated questionnaires to evaluate symptoms associated with visual dysfunctions have been used in combination with a complete battery of tests in order to provide a comprehensive analysis of changes occurring after vision therapy.

### 4.5. Changes in Stereopsis

In the present study, stereopsis did not show a statistically significant improvement. Although mean stereoacuity decreased slightly from 557 to 540 s of arc, the high interindividual variability (large standard deviation and wide ranges) suggests that this parameter was less sensitive to the type of training applied. Nevertheless, it should be considered that many of the patients evaluated had stereoacuity within normal limits for their age. CI does not always affect stereopsis, as it does not always completely disrupt binocularity, and some level of fusion and stereopsis may still exist. Future studies should investigate this aspect further in order to understand the real changes occurring in PD patients after digital visual training.

### 4.6. Compliance

Developing this study has posed a significant methodological and logistical challenge, largely due to the characteristics of the research population: patients with PD who, in addition to their motor difficulties, frequently present associated cognitive impairments. This clinical profile implies that participants not only face physical limitations, such as tremors, rigidity, or bradykinesia, but also cognitive difficulties affecting working memory, attention, planning, and motivation. These conditions directly impact their ability to follow instructions, maintain consistency in a training routine, and autonomously use the digital platform evaluated. One of the main challenges has been ensuring adherence to the prescribed visual therapy, as many patients require constant support from family members or caregivers to complete the sessions. In several cases, technical difficulties, lack of digital skills, or the patient’s own lack of motivation posed significant obstacles. This forced the research team to establish personalized follow-up strategies, maintain continuous communication with caregivers, and adjust timelines and expectations according to each subject’s individual pace. Even with these measures, the variability in the degree of compliance was notable, as reflected by the data collected on platform usage. Specifically, mean play game time was 135.5 min in our series, with a median of 104.5, indicating a slightly asymmetric distribution. The mean adherence was 37.6%, which, although it could be considered moderate, allowed for the observation of clinically significant effects on various parameters. There were some extreme specific cases, with two subjects reaching compliance rates of more than 100% because they practiced even on the weekly rest day for the therapy, and one patient with a compliance rate of 0.8%. The limited previous research on vision therapy for the management of CI has also evidenced the problem of obtaining acceptable compliance when performing in-person visual training sessions combined with home reinforcement. Kergoat et al. [15] reported difficulties of the PD population in completing traditional visual treatments: of the 25 patients diagnosed with CI, only 7 agreed to start therapy and only 2 completed it. Reasons included fatigue, physical limitations, low motivation, and caregiver dependence. For this reason, protocols of vision therapy used in neurologically healthy children or adults cannot be directly extrapolated to the PD population, making necessary the development of specific therapeutic approaches.

A clear correlation was found between the level of compliance and the improvement observed in some parameters, including symptomatology and NPC. This confirms the necessity of maintaining a good treatment adherence and the need for maintaining an online control of the sessions performed by the patient in order to detect any event suggesting an interruption of the therapeutic process. In this sense, it is worth noting that gamified, video game-based rehabilitation has been shown to be a feasible and engaging strategy, with acceptable adherence levels and clinically relevant benefits, suggesting that further optimization of game design and support strategies could help maximize compliance in PD patients as well [45].

### 4.7. Limitations

Several methodological limitations have been identified that should be considered when interpreting the results. Of note is the possible presence of uncontrolled confounding variables, such as disease duration, medication taken by patients, or degree of motor impairment. These factors could have modulated the relationship between CI and PD, complicating the causal interpretation of the findings. In addition, the lack of a control group and the limited sample size are important constraints that should be acknowledged. Future randomized controlled trials should be conducted to confirm the outcomes of this preliminary study. However, the definition of the control group must be approached with care, as withholding vision therapy from a specific group upon detection of symptomatic CI in patients with PD raises ethical concerns. Alternatively, the control group could comprise patients treated with a conventional protocol of office-based vision therapy with home reinforcement.

The treatment duration represents a further limitation, as it could be extended in future studies in order to confirm whether the improvements achieved could be even greater. Likewise, the absence of objective measures of ocular motility, such as eye tracking, constitutes an additional limitation; however, two subjective tests were employed instead, which have shown comparable outcomes. Future trials evaluating the effectiveness of this digital system should incorporate this methodological aspect.

Finally, the use of different smartphone and tablet devices may have introduced some level of additional variability due to potential inter-subject differences in terms of visual angle and extent of peripheral stimulation during treatment. Likewise, viewing distance, which can affect vergence demand, could not be monitored as this would have introduced some additional variability due to potential variations between participants and each individual session. Future developments for avoiding this include continuous monitoring of the viewing distance through the frontal camera within the app.

Despite these limitations, the present study represents the first scientifically valid and clinically applicable investigation demonstrating that a video game-based vision therapy intervention can significantly improve vergence function, oculomotor control, and symptom perception in patients with PD and CI. Unlike the classic approach, this digital modality offers advantages in accessibility, personalization, and monitoring, which may facilitate its integration into neurological rehabilitation programs. These results therefore not only complement existing findings in the literature but also position this intervention as a viable and promising alternative warranting further exploration in future research with larger samples and longitudinal follow-up.

## 5. Conclusions

The use of a digital tool specifically designed for visual rehabilitation in patients with CI appears to be effective in improving various aspects of visual function, even when therapeutic adherence does not reach optimal levels, in patients with PD and associated CI. It should be noted that this is a pilot study and its results cannot be generalized to the broader PD population; therefore, further investigations are warranted.

## Figures and Tables

**Table 1:** Summary of data concerning the game performance and compliance with treatment.

	Mean (Standard Deviation)	Median	Range
Game scoring	2618.5 (2149.5)	2122.5	0 to 7449
Total minutes of play	135.5 (124.6)	104.5	3 to 449
Compliance (%)	37.6 (34.6)	29.0	0.8 to 124.7

**Table 2:** Summary of data concerning the fusional vergences measured at near. Abbreviations: PFV, positive fusional vergence; NFV, negative fusional vergence; NPC, near point of convergence.

		Mean (Standard Deviation)	Median	Range (Min to Max)	*p*-Value	Effect Size
Break NPC	Pre-therapy	15.2 (5.8)	14.0	8 to 25	0.022	0.87
	Post-therapy	6.5 (7.5)	4.0	0 to 25		
Recovery NPC	Pre-therapy	21.2 (6.2)	17.0	16 to 30	0.007	1.34
	Post-therapy	6.8 (6.3)	6.0	0 to 16		
Break NFV	Pre-therapy	6.9 (4.8)	6.0	1 to 18	0.108	−0.60
	Post-therapy	12.7 (5.5)	12.0	6 to 25		
Recovery NFV	Pre-therapy	4.8 (4.7)	4.0	0 to 16	0.126	−0.57
	Post-therapy	10.0 (5.3)	8.0	2 to 20		
Break PFV	Pre-therapy	11.5 (7.1)	10.0	2 to 15	0.003	−1.30
	Post-therapy	22.7 (7.9)	25.0	10 to 35		
Recovery PFV	Pre-therapy	7.7 (6.8)	4.0	0 to 20	<0.001	−2.14
	Post-therapy	17.5 (6.9)	20.0	4 to 30		

**Table 3:** Summary of data from the oculomotricity tests NSUCO and King-Devick (K-D).

		Mean (Standard Deviation)	Median	Range	*p*-Value	Effect Size
NSUCO score pursuits	Pre-therapy	11.6 (2.0)	12.0	7 to 15	0.006	−0.89
	Post-therapy	12.9 (2.1)	13.0	8 to 15		
NSUCO score saccades	Pre-therapy	12.3 (2.2)	13.0	7 to 15	0.008	−0.84
	Post-therapy	13.4 (1.8)	13.5	9 to 15		
NSUCO total score	Pre-therapy	23.9 (4.2)	25.0	14 to 30	0.003	−0.98
	Post-therapy	26.2 (3.7)	26.0	17 to 30		
K-D time card I	Pre-therapy	27.7 (11.4)	23.0	15 to 53	0.018	0.72
	Post-therapy	23.3 (7.3)	21.5	14 to 38		
K-D time card II	Pre-therapy	24.7 (8.7)	21.0	14 to 43	0.293	0.29
	Post-therapy	22.7 (7.9)	20.5	13 to 42		
K-D time card III	Pre-therapy	27.2 (10.9)	23.5	15 to 52	0.053	0.57
	Post-therapy	22.9 (7.2)	20.0	14 to 39		
K-D test failures	Pre-therapy	4.4 (11.4)	0.0	0 to 9	0.427	0.29
	Post-therapy	5.2 (15.9)	0.0	0 to 7		
Total K-D time	Pre-therapy	79.4 (28.8)	72.0	44 to 143	0.034	0.63
	Post-therapy	69.0 (21.5)	60.5	41 to 115		

## Data Availability

Dataset available on reasonable request from the authors.
